# Self-Aligned Top-Gate Metal-Oxide Thin-Film Transistors Using a Solution-Processed Polymer Gate Dielectric

**DOI:** 10.3390/mi11121035

**Published:** 2020-11-25

**Authors:** Seungbeom Choi, Seungho Song, Taegyu Kim, Jae Cheol Shin, Jeong-Wan Jo, Sung Kyu Park, Yong-Hoon Kim

**Affiliations:** 1 School of Advanced Materials Science and Engineering, Sungkyunkwan University, Suwon 16419, Korea; csb881001@gmail.com (S.C.); play236@naver.com (S.S.); 68kevin@hanmail.net (T.K.); 2 School of Electrical and Electronic Engineering, Chung-Ang University, Seoul 06980, Korea; tlswo0627@naver.com; 3 Department of Electrical Engineering, University of Cambridge, Cambridge CB2 1TN, UK; jzw0108@gmail.com; 4 SKKU Advanced Institute of Nanotechnology (SAINT), Sungkyunkwan University, Suwon 16419, Korea

**Keywords:** self-aligned, top-gate, thin-film transistor, solution process, polymer gate dielectric

## Abstract

For high-speed and large-area active-matrix displays, metal-oxide thin-film transistors (TFTs) with high field-effect mobility, stability, and good uniformity are essential. Moreover, reducing the RC delay is also important to achieve high-speed operation, which is induced by the parasitic capacitance formed between the source/drain (S/D) and the gate electrodes. From this perspective, self-aligned top-gate oxide TFTs can provide advantages such as a low parasitic capacitance for high-speed displays due to minimized overlap between the S/D and the gate electrodes. Here, we demonstrate self-aligned top-gate oxide TFTs using a solution-processed indium-gallium-zinc-oxide (IGZO) channel and crosslinked poly(4-vinylphenol) (PVP) gate dielectric layers. By applying a selective Ar plasma treatment on the IGZO channel, low-resistance IGZO regions could be formed, having a sheet resistance value of ~20.6 kΩ/sq., which can act as the homojunction S/D contacts in the top-gate IGZO TFTs. The fabricated self-aligned top-gate IGZO TFTs exhibited a field-effect mobility of 3.93 cm^2^/Vs and on/off ratio of ~10^6^, which are comparable to those fabricated using a bottom-gate structure. Furthermore, we also demonstrated self-aligned top-gate TFTs using electrospun indium-gallium-oxide (IGO) nanowires (NWs) as a channel layer. The IGO NW TFTs exhibited a field-effect mobility of 0.03 cm^2^/Vs and an on/off ratio of >10^5^. The results demonstrate that the Ar plasma treatment for S/D contact formation and the solution-processed PVP gate dielectric can be implemented in realizing self-aligned top-gate oxide TFTs.

## 1. Introduction

Amorphous metal-oxide semiconductor-based thin-film transistors (TFTs) have gathered significant interest in active-matrix-driven displays such as organic light-emitting diodes and liquid crystal displays due to their outstanding electrical performance such as high field-effect mobility, low off-state current and excellent electrical stability [1,2,3]. In addition, their amorphous nature can offer advantages such as high spatial uniformity over a large area, which allows the demonstration of large-size electronics. Despite the advantages of amorphous oxide-based TFTs, the use of the conventional bottom-gate structure often results in the formation of high parasitic capacitance due to the overlap between the source/drain (S/D) and the gate electrodes [4]. This can cause a significant RC delay during the operation of the displays, resulting in degradation of the image quality. To resolve this issue, specifically to reduce the effects of parasitic capacitance, a self-aligned top-gate structure has been adopted in oxide TFTs [5]. By using the self-aligned structure, the overlapped area between the gate and S/D electrodes can be significantly reduced, resulting in a decrease in parasitic capacitance as well as the RC delay.

To realize the self-aligned top-gate oxide TFTs, the S/D contact regions in IGZO channel should have high electrical conductivity. Previously, various doping elements such as aluminum and fluorine have been investigated [6,7]. By doping these elements into the metal contact regions of IGZO film, the electrical conductivity can be significantly increased, allowing low-contact resistance with the S/D metals. Although doping these metallic or halogen atoms is effective in increasing the conductivity, a sophisticated ion implantation equipment is typically required to dope the elements into the oxide channel layer. Additionally, a hydrogen-doping process was also suggested to form the S/D contact regions [8]. However, the hydrogen atoms are highly mobile and rather unstable at high temperatures [9]. Therefore, controlling the concentration of hydrogen may be difficult, especially when using a high process temperature. Compared to these approaches, a simple argon (Ar) plasma treatment can also be used to form the S/D contact regions [10]. Particularly, by the Ar plasma treatment, the oxygen vacancy concentration in the exposed regions can be increased, which leads to an increase in electrical conductivity. Moreover, the Ar plasma process requires relatively simple process equipment compared to other doping methods and only requires a short process time to obtain high electrical conductivity [11]. Therefore, by optimizing the Ar plasma process, self-aligned top-gate oxide TFTs can be simply realized.

Furthermore, solution processing of oxide channel as well as the gate dielectric can offer potential advantages such as the non-vacuum and scalable fabrication of high-performance oxide TFTs. Particularly, for the gate dielectric layer, the utilization of insulating polymers can be effective in simplifying the fabrication process and lowering the process temperature [12]. Due to the low Young’s modulus of the polymers, the mechanical flexibility of the TFTs can be improved [13]. Among the various polymer insulators, crosslinked poly(4-vinylphenol) (PVP) gate dielectric has been widely studied in organic TFT devices owing to its relatively high dielectric constant and good electrical stability [14]. The PVP gate dielectric can be solution-processed at a relatively low temperature, typically below 200 °C.

Here, we report self-aligned top-gate oxide TFTs using solution-processed indium-gallium-zinc-oxide (IGZO) channel and crosslinked PVP gate dielectric layers. For the realization of self-aligned top-gate structure, a selective Ar plasma treatment was applied at the S/D contact regions of the IGZO channel. With optimized Ar plasma treatment condition, the sheet resistance of IGZO film could be reduced to ~20.6 kΩ/sq. The fabricated self-aligned top-gate IGZO TFTs exhibited a field-effect mobility of 3.93 cm^2^/Vs and on/off ratio of ~10^6^. Additionally, we also demonstrate self-aligned top-gate TFTs using electrospun indium-gallium-oxide (IGO) nanowires (NWs) as a channel layer. The fabricated IGO NW TFTs exhibited a field-effect mobility of 0.03 cm^2^/Vs and on/off ratio of >10^5^. The results shown that the Ar plasma treatment for S/D contact formation and the solution-processed PVP gate dielectric can be implemented in realizing self-aligned top-gate oxide TFTs.

## 2. Experimental Procedure

To prepare the IGZO precursor solution, indium nitrate hydrate, gallium nitrate hydrate and zinc nitrate hydrate were dissolved in 2-methoxyethanol at a total concentration of 0.125 M. Before use, the solution was thoroughly stirred at 75 °C for around 24 h. The molar ratio of In:Ga:Zn was 6.8:1.0:2.2. To fabricate the self-aligned top-gate IGZO TFTs, the IGZO precursor solution was spin-coated on a Si/SiO_2_ wafer and thermal annealed at 350 °C for 1 h. The IGZO channel was then patterned by photolithography and wet etching. For the gate dielectric, a PVP solution was first prepared by dissolving PVP powder, poly(melamine-co-formaldehyde) in propylene glycol monomethyl ether acetate with a weight ratio of 10:5:85, respectively. After stirring, the PVP solution was spin-coated onto the patterned IGZO layer and baked at 150 °C for 30 min. The thickness of PVP layer was ~718 nm, which was measured by a surface profiler (alpha-step IQ, KLA Tencor, Milpitas, CA, USA). Then, Cr was deposited as a top gate electrode with a thickness of 50 nm. The patterning of Cr gate electrode was carried out by using a metal shadow mask. To etch the PVP gate dielectric, an O_2_ plasma process was carried out at 100 W for 3 min. During the PVP etching process, the Cr gate electrode worked as a screening mask for patterning the underneath PVP layer. Afterward, to form S/D contact regions, an Ar plasma process was performed at 50 W for 0~180 s (Asher, RIE system, SNTEK Co., Ltd., Suwon, Korea).

For the fabrication of IGO NW TFTs, an electrospinning process was carried out. First, an IGO precursor solution was prepared by dissolving indium nitrate hydrate and gallium nitrate hydrate in a solution containing 0.7 g of polyvinylpyrrolidone (M_w_~1,300,000) and 5 mL of N,N-dimethylformamide (DMF). The mixed solution was then stirred for 24 h at room temperature. To fabricate the TFTs, the IGO precursor solution was electrospun on a Si/SiO_2_ wafer (ESR200R2, NanoNC, Seoul, Korea). The solution was ejected from a needle (23 gauge) at an electrical field of 13 kV for 15 s. The distance between the needle and the substrate was around 15 cm. The ejected NW composite was baked at 150 °C, and then finally annealed at 450 °C for 2 h to remove polyvinylpyrrolidone and other organic components in the solvent. The rest of the TFT fabrication processes were identical to the IGZO TFTs.

To measure the sheet resistance of the Ar plasma-treated IGZO films, IGZO films were coated on bare glass substrates. The sheet resistance was measured using a 4-point probe measurement system (CMT-SR1000N, AIT, Suwon, Korea). To evaluate the change in the oxygen binding states in IGZO films, depending on the Ar plasma time, X-ray photoelectron spectroscopy (XPS) analysis was carried out (ESCALAB 250, Thermo scientific, Rockford, IL, USA). The capacitance of the PVP gate dielectric was measured by using a precision LCR meter (Agilent 4284A) and the leakage current density of PVP gate dielectric and the electrical characteristics of IGZO and IGO NW TFTs were analyzed using a semiconductor parameter analyzer (Agilent 4155C). The microstructure of IGO NW channels was analyzed using field emission scanning electron microscopy (FESEM; JSM-7600F, JEOL, Tokyo, Japan).

## 3. Results and Discussion

**Figure 1:**
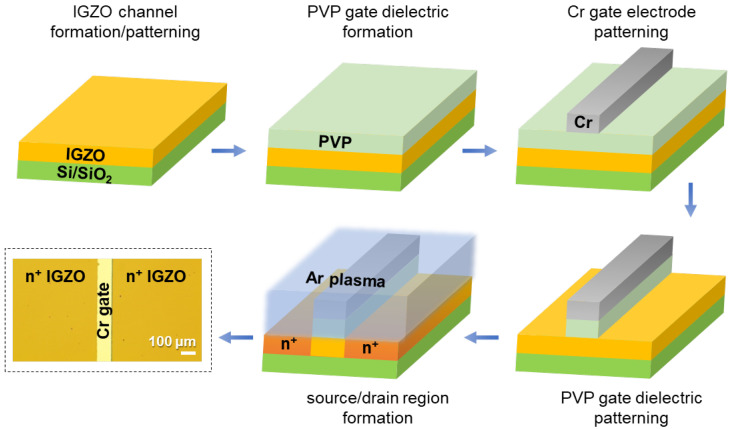
Fabrication process, schematic device structure and an optical image of self-aligned top-gate indium-gallium-zinc-oxide (IGZO) thin-film transistors (TFTs). The deposition of IGZO channel and poly(4-vinylphenol) (PVP) gate dielectric layers was carried out by using a solution process. The Ar plasma treatment was utilized to form the low-resistance S/D contact regions.

**Figure 2:**
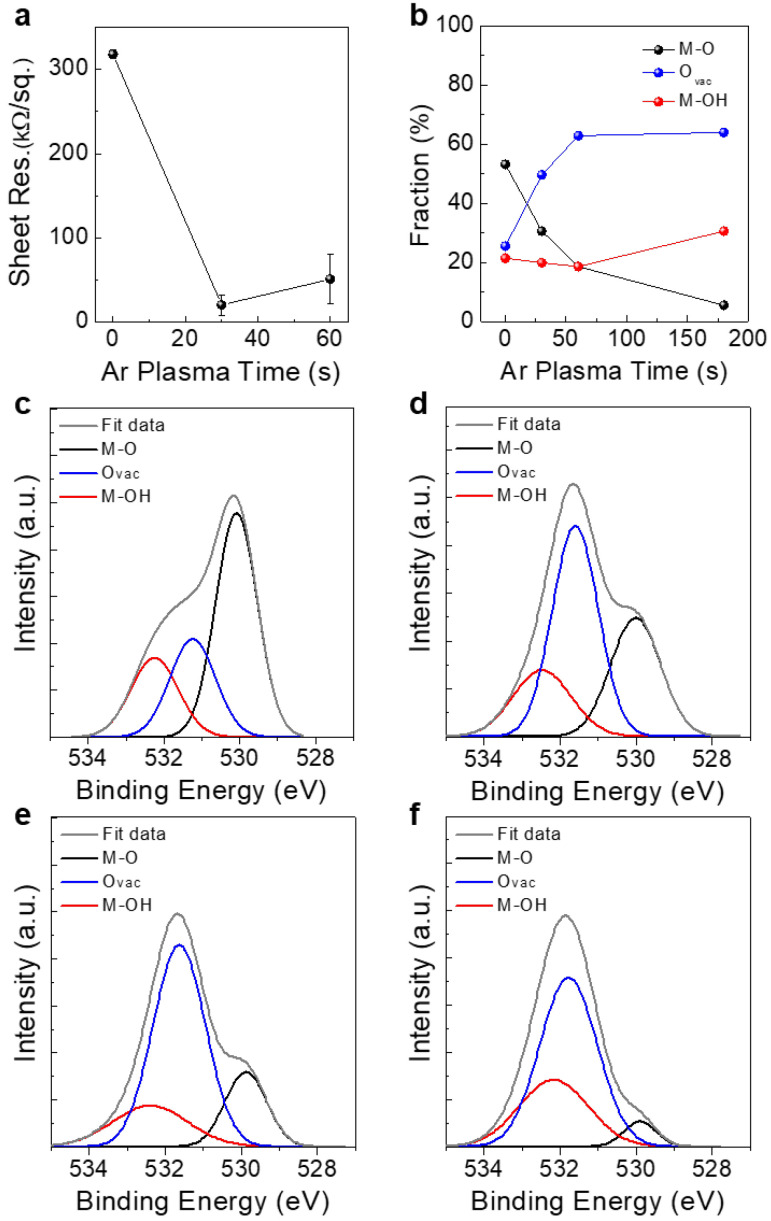
(**a**) Sheet resistance variation of IGZO films as a function of Ar plasma time. (**b**) The change in metal–oxygen (M-O), oxygen vacancy (O_vac_), and metal hydroxide (M-OH) binding states in IGZO films as a function of Ar plasma time. O 1s peaks obtained from IGZO films with Ar plasma time of (**c**) 0 s, (**d**) 30 s, (**e**) 60 s, and (**f**) 180 s.

The fabrication process and schematic device structure of self-aligned top-gate IGZO TFTs are shown in Figure 1. As described, the PVP gate dielectric is patterned using O_2_ plasma etching using Cr gate electrode as a screening mask. Under the etched PVP layer, IGZO regions for S/D contacts are then exposed. However, due to the low electrical conductivity of the intrinsic IGZO film, high contact resistance and, subsequently, a large voltage drop can occur at these regions. To increase the conductivity of the IGZO film and to build a homojunction channel structure, an Ar plasma treatment was carried out. Here, due to the Cr gate and PVP gate dielectric layers, only the S/D contact regions are exposed to Ar plasma, as shown in Figure 1. To investigate the effect of Ar plasma treatment on the electrical conductivity of IGZO film, Ar plasma time was varied from 0 to 180 s. Figure 2a shows the variation in sheet resistance as a function of Ar plasma time. The intrinsic IGZO film without the Ar plasma treatment exhibited a sheet resistance of 317.9 kΩ/sq., which decreased to 20.6 kΩ/sq. and 51.1 kΩ/sq. after 30 and 60 s of Ar plasma treatment, respectively. The main reason for the resistance decrease by the Ar plasma treatment can be ascribed to the formation of oxygen vacancies in the IGZO film [9]. Specifically, the energetic Ar^+^ bombardment during the plasma process can induce preferential sputtering of relatively lighter atoms from the film surface, such as the oxygen atoms in the IGZO film [10]. The relatively heavier atoms such as indium, gallium and zinc have a lower sputtering yield, therefore, oxygen vacancies are preferentially formed near the surface region. By the formation of oxygen vacancies, excess free electrons are generated in the film, which increase the carrier concentration and the conductivity of IGZO film [15]. Interestingly, the sheet resistance was saturated at around 30~60 s of Ar plasma treatment time. This can be attributed to the reaching of a steady-state condition at which the sputtering yield of oxygen atoms balances those of metal atoms in the oxygen-deficient IGZO region [10]. However, by increasing the Ar plasma treatment time up to 180 s, the sheet resistance could not be measured, mainly due to the high resistance of the IGZO film.

To investigate the influence of Ar plasma treatment on the oxygen-binding states of IGZO film and the formation of oxygen vacancies, an XPS analysis was carried out. Figure 2b shows the variation in metal–oxygen (M-O), oxygen vacancy (O_vac_), and metal hydroxide (M-OH) binding states in IGZO films as a function of Ar plasma time. Figure 2c–f also shows the series of O 1s peaks obtained from IGZO films treated with different Ar plasma timea. Here, the O 1s peaks are de-convoluted into three main peaks, M-O, O_vac_ and M-OH. The peaks centered at around 530, 531 and ~532 eV correspond to M-O, O_vac_ and M-OH binding states, respectively. Overall, as the Ar plasma time increased, the portion of M-O states was significantly decreased from 53.2% to 5.5%, while the portions of O_vac_ and M-OH states increased from 25.5% to 64.0% and from 21.4% to 30.6%, respectively. As aforementioned, a continuous exposure to Ar plasma can induce a substantial increase in oxygen vacancies which can contribute to an increase in electrical conductivity. Therefore, the abrupt decrease in sheet resistance observed at the Ar plasma time of 30~60 s can be attributed to the generation of significant amounts of oxygen vacancies in the IGZO film. Interestingly, the sheet resistance was slightly higher when the Ar plasma time was increased from 30 to 60 s. It was also observed that a prolonged Ar plasma treatment (180 s) induced a high increase in sheet resistance (not measurable by the 4-point probe method). It is speculated that the significant generation of defective M-OH binding states, as well as a substantial decrease in M-O binding states, are responsible for the increase in sheet resistance.

**Figure 3:**
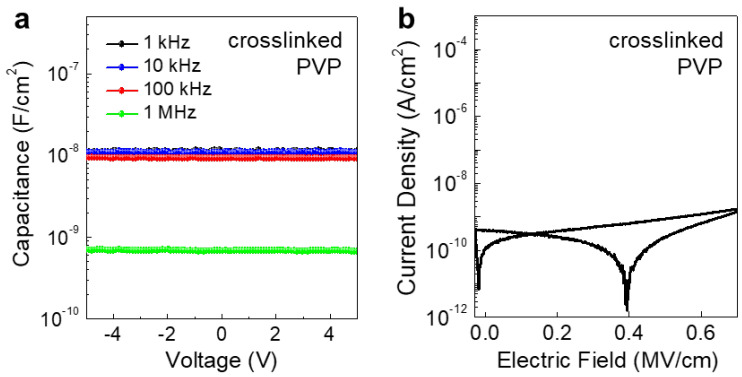
(**a**) Capacitance vs. frequency, and (**b**) the current density vs. electric field (J-E) characteristics of crosslinked PVP gate dielectric layer. The thickness of the PVP gate dielectric layer was ~718 nm.

For the self-aligned top-gate IGZO TFTs, a crosslinked PVP gate dielectric was used. The PVP gate dielectric has been used in organic TFTs owing to its high dielectric constant, low leakage current, and low-temperature solution processability [16]. To investigate the dielectric properties of the crosslinked PVP gate dielectric layer, the capacitance vs. frequency and the current density vs. electric field (J-E) were analyzed. As shown in Figure 3a, the PVP gate dielectric exhibited relatively stable capacitance characteristics up to 1 MHz, with a relatively large decrease in capacitance at 1 MHz. It is reported that the unreacted hydroxyl groups remaining in the PVP gate dielectric can be responsible for the frequency-dependent capacitance variation [17]. Particularly, the unreacted hydroxyl groups can easily attract the water molecules and mobile ions, which results in slow polarization and low capacitance value at high frequencies. Furthermore, the J-E characteristics are shown in Figure 3b. The PVP gate dielectric exhibited a low leakage current density of <2 × 10^−9^ A/cm^2^ up to an electric field of ~0.7 MV/cm. Although some hysteresis behaviors were observed during the double sweep measurement, the overall current density values were low, in the range of 10^−9^~10^−12^ A/cm^2^. Nonetheless, the results of capacitance and the leakage current density show that the crosslinked PVP film can be a possible candidate for a gate dielectric in top-gate oxide TFTs.

**Figure 4:**
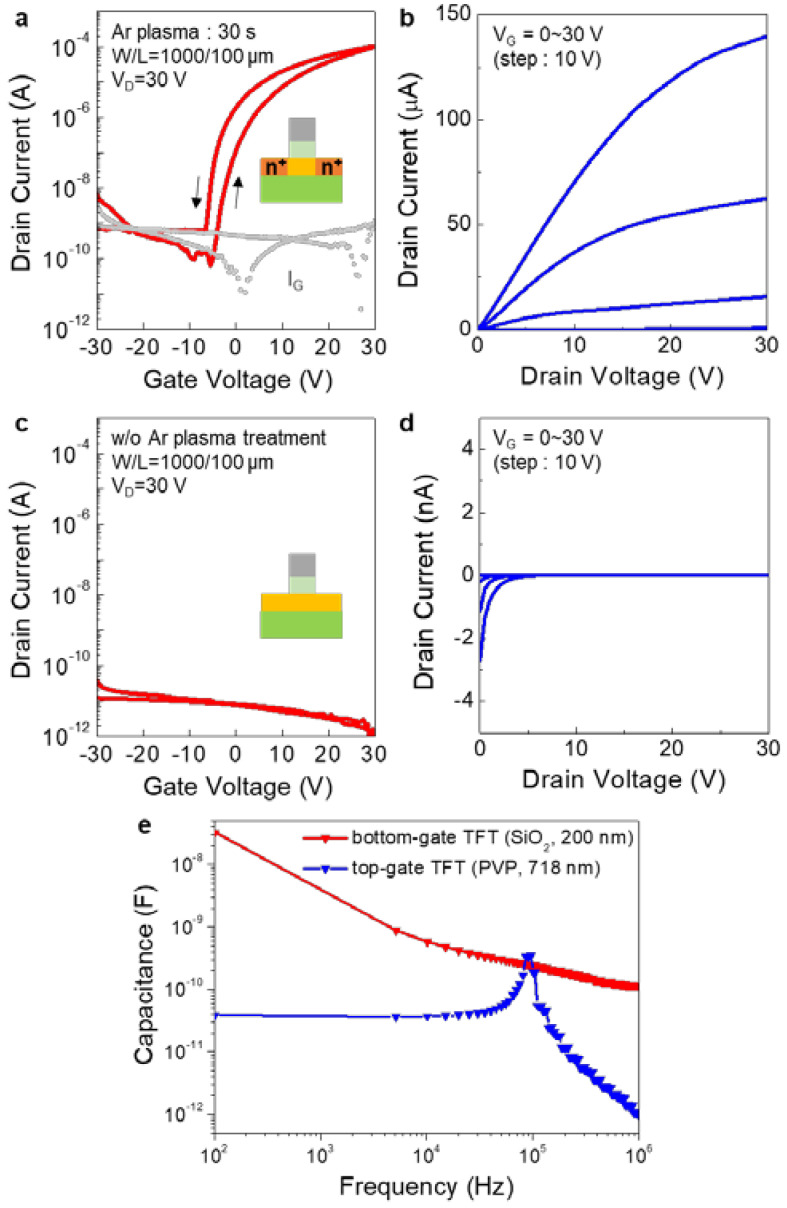
(**a**) Transfer and (**b**) output characteristics of self-aligned top-gate IGZO TFTs fabricated with Ar plasma treatment time of 30 s. The channel width and length were 1000 and 100 µm, respectively. (**c**) Transfer and (**d**) output characteristics of self-aligned top-gate IGZO TFTs fabricated without the Ar plasma treatment. In this case, the TFTs showed no switching behavior. (**e**) Capacitance-frequency data obtained from bottom-gate IGZO TFTs using SiO_2_ gate dielectric (thickness ~200 nm) and top-gate IGZO TFTs using PVP gate dielectric (thickness ~718 nm).

Using the solution-processed PVP gate dielectric, self-aligned top-gate IGZO TFTs were fabricated and their electrical characteristics were measured. Figure 4a,b shows the transfer and output characteristics of the top-gate IGZO TFTs, respectively, having a channel width (W) of 1000 µm and channel length (L) of 100 µm. In this case, the Ar plasma treatment for the S/D contact formation was performed for 30 s, which exhibited the lowest sheet resistance (Figure 2a). As shown in Figure 4a, the top-gate IGZO TFTs exhibited a decent switching behavior with a current on/off ratio of ~10^6^ and threshold voltage (V_th_) of 4.04 ± 1.53 V. The saturation field-effect mobility (μ) was calculated using the following equation

(1) $$I_{D}=\frac{W}{2L}{\mu C}_{i}{\left(V_{G}-V_{\mathrm{th}}\right)}^{2}$$

where I_D_ is the drain current, L and W are the channel length and width, respectively, C_i_ is the areal capacitance of the gate dielectric, and V_g_ is gate voltage. The saturation field-effect mobility was 3.93 ± 0.22 cm^2^/Vs which is comparable to those fabricated with SiO_2_ gate dielectric layer [18]. On the other hand, without the Ar plasma treatment, the device showed no switching behavior or charge accumulation. As shown in Figure 4c,d, the drain current (I_D_) was not varied by the gate bias (V_G_) and remained at current levels of 10^−11^~10^−12^ A. This can be attributed to the high resistivity of the S/D contact regions in the IGZO channel. Due to the high resistance in these regions, a substantial voltage drop can occur at these contacts, which decreases the effective bias applied at the channel region [19]. As a result, the drain current level can be comparably lower than the devices with low-resistance S/D contact regions. In addition, the parasitic capacitance of the self-aligned top-gate IGZO TFTs were analyzed in the range of 10^2^~10^6^ Hz. As shown in Figure 4e, even though the PVP has slightly lower dielectric constant (ε~3.6) than the SiO_2_ (ε~3.9), and thicker than the SiO_2_ gate dielectric (thickness ~200 nm), the parasitic capacitance values measured in top-gate IGZO TFTs were comparably lower than that of the bottom-gate IGZO TFTs in most of the frequency range. Nonetheless, these results clearly show that the Ar plasma treatment is effective in lowering the electrical resistivity of IGZO channel at the S/D contact regions, which is required to operate the self-aligned top-gate oxide TFTs.

**Figure 5:**
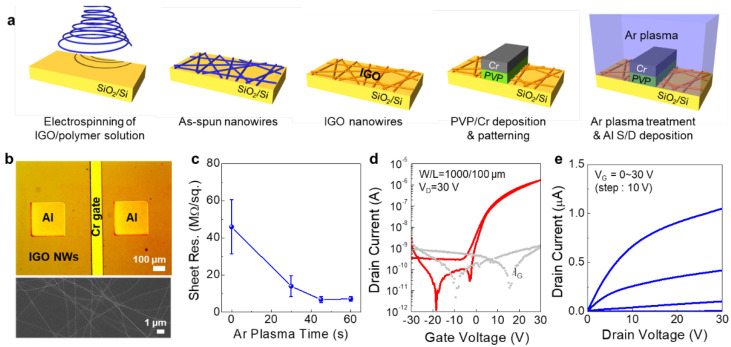
(**a**) Fabrication process of self-aligned top-gate IGO NW TFTs. The IGO NW channel was fabricated using an electrospinning process. (**b**) An optical image of IGO NW TFT and a FESEM image of the IGO NW channel. (**c**) The variation of IGO NW film as a function of Ar plasma time. (**d**) Transfer and (**e**) output characteristics of self-aligned top-gate IGO NW TFTs fabricated with Ar plasma treatment time of 30 s. The channel width and length of 1000 µm and 100 µm, respectively.

The strategy of using Ar plasma treatment and PVP gate dielectric for self-aligned top-gate oxide TFTs has been further applied to oxide NW TFTs. Here, electrospun IGO NWs were adopted as a channel layer, as shown in Figure 5a. After electrospinning and annealing the IGO NWs, a PVP gate dielectric was spin-coated over the IGO NW channel. Afterward, deposition and patterning of Cr gate electrode, and Ar plasma treatment for S/D contact formation were carried out. Additionally, as S/D electrodes, aluminum electrode was deposited over the IGO NWs. Figure 5b shows an optical image of the fabricated self-aligned top-gate IGO NW TFTs. The FESEM image of the IGO NW channel is also displayed. The sheet resistance variation in the IGO NW film was evaluated for different Ar plasma treatment times, as shown in Figure 5c. Before the Ar plasma, the IGO NW film exhibited a sheet resistance of 45.9 MΩ/sq., while it gradually decreased to 7.2 MΩ/sq. when the Ar plasma time was increased to 60 s. The sheet resistance value was also saturated after around 30 s, similar to that observed in IGZO films. Figure 5d,e show the transfer and output characteristics of the self-aligned top-gate IGO NW TFTs with an Ar plasma time of 30 s. The device showed good switching behavior with an on/off ratio of 10^5^~10^6^, V_th_ of 9.58 ± 0.53 V and field-effect mobility of 0.03 ± 0.005 cm^2^/Vs. The relatively low mobility compared to the IGZO TFTs can be attributed to the low area coverage of IGO NWs in the channel region. Compared to thin-film type channels, the IGO NWs only cover partial areas of the channel region in which the electrons are transported. For the calculation of field-effect mobility using Equation (1), we used the dimension of the IGO NW channel (W = 1000 µm). Nonetheless, the results also show that the Ar plasma treatment for S/D contact formation and PVP gate dielectric can be used to realize oxide NW-based TFTs.

**Figure 6:**
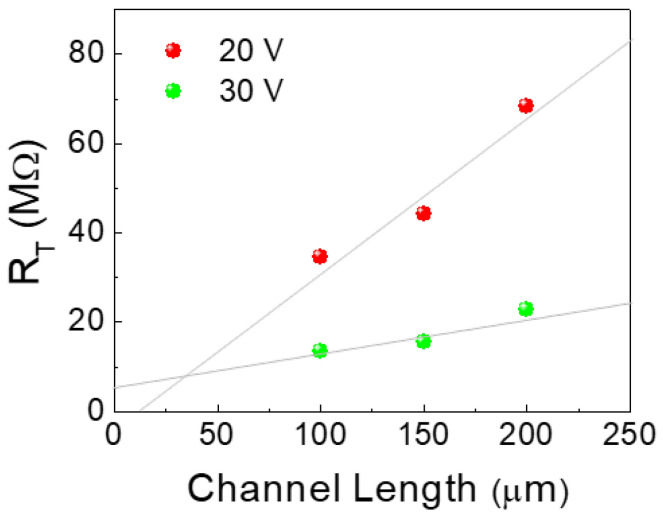
Total resistance vs. channel length (R_T_-L) plots obtained from IGO NW TFTs having channel lengths of 100~200 µm (V_G_ = 20 V, 30 V).

To further investigate the influence of Ar plasma treatment on the semiconductor/metal contact properties in IGO NW TFTs, the contact resistance was calculated using the transmission line method (TLM) [20]. As described, excess electrons are generated by the formation of oxygen vacancies during the Ar plasma treatment. In addition, by the Ar^+^ bombardment, some heat can be generated on the IGO NW surface, which, in turn, induces some electrons to diffuse into the IGO channel region below the Cr/PVP layers, forming a parasitic resistance [21]. Here, the effective channel length (L_eff_) can be calculated by subtracting the diffused regions (ΔL) from the actual channel length (L). Accordingly, the total resistance of the channel (R_T_) can be expressed as the following [22,23,24]

(2) $$R_{T}=\frac{L-2\Delta L}{\mu_{i}{\mathrm{WC}}_{\mathrm{ox}}\left(V_{G}-V_{\mathrm{th}}\right)}+2R_{0}$$

where µ_i_ is the intrinsic field-effect mobility, C_ox_ is the areal capacitance, V_th_ is the threshold voltage, and R_0_ is the parasitic resistance which represents the limit of S/D series resistance for high gate voltages and is related to the S/D contact resistance [23]. Figure 6 shows the R_T_-L plots obtained from the IGO NW TFTs, having channel lengths of 100~200 µm (V_G_ = 20 V, 30 V). Here, the crosspoint (x,y) made between the two fitted lines indicates x = 2·ΔL and y = 2·R_0_. From the plots, the extracted values of ΔL and width-normalized parasitic resistance (R_0_·W) were ~13.5 μm and 350 kΩcm, respectively. This indicates that some conductive regions may exist below the Cr gate electrode, which should be reduced to minimize the effects of parasitic capacitance.

## 4. Conclusions

In this paper, we demonstrated self-aligned top-gate oxide TFTs using solution-processed IGZO channel and crosslinked PVP gate dielectric layers. By applying a selective Ar plasma treatment on the IGZO channel, homojunction S/D contact regions with a low electrical resistance could be formed. It was found that the Ar plasma treatment induced the generation of oxygen vacancies in the IGZO channel layer, which increased the conductivity. Using the Ar plasma process, self-aligned top-gate IGZO and IGO NW TFTs exhibiting proper switching behavior and decent field-effect mobility and on/off ratio were realized. The results show that the Ar plasma treatment for S/D contact formation and solution-processed PVP gate dielectric can be successfully implemented in realizing self-aligned top-gate oxide TFTs.
